# Health-related quality of life among patients with cirrhosis at a tertiary care center in Addis Ababa, Ethiopia: a cross-sectional study

**DOI:** 10.3389/fgstr.2024.1455837

**Published:** 2024-12-12

**Authors:** Edgeit Abebe Zewde, Awgichew Behaile Teklemariam, Tesfaneh Shemeles Ayele, Melaku Mekonnen Agidew, Anemut Tilahun Mulu, Metages Damtie Melaku

**Affiliations:** ^1^ Department of Biomedical Sciences, College of Health Sciences, Debre Tabor University, Debre Tabor, Ethiopia; ^2^ Department of Public Health, College of Health Sciences, Debre Tabor University, Debre Tabor, Ethiopia; ^3^ Department of Internal Medicine College of Health Sciences, Debre Tabor University, Debre Tabor, Ethiopia

**Keywords:** cirrhosis, Child-Turcotte-Pugh (CTP) classification, health-related quality of life, Ethiopia, MUAC (Mid-Upper Arm Circumference)

## Abstract

**Background:**

Health-related quality of life (HRQoL) reflects an individual’s perception of how disease and treatment affect their physical, functional, emotional, and social well-being. The burden of cirrhosis is high in Ethiopia but reports on HRQoL among cirrhosis patients are lacking. This study aimed to assess HRQoL and associated factors among patients with cirrhosis at Tikur Anbessa Specialized Hospital, Addis Ababa, Ethiopia.

**Methods:**

A cross-sectional study was conducted among 221 patients with cirrhosis at Tikur Anbessa Specialized Hospital. Data were coded and entered into Epi Data 4.6.0.2 and analyzed by STATA software version 17m/p. Bivariable and multivariable linear regression analyses were performed. A p-value lower than 0.05 was used to declare statistical significance.

**Results:**

The mean ± SD age was 42.74 ± 12.25. In multivariable linear regression analysis, Mid Upper Arm Circumference (MUAC) (Standardized β.coff (Std.β.coff) = 0.12, 95% CI (0.06, 0.18), age ≥50 (Std.β.coff = -0.38, 95% CI (-0.68,-0.09), Child-Turcotte-Pugh (CTP) class B (Std.β.coff = -0.92, 95% CI (-1.28, -0.55), CTP class C (Std.β.coff = -1.69, 95% CI (-2.30,-1.07) were significantly associated with mean quality of life score.

**Conclusion:**

Quality of life score was significantly associated with age, CTP class, and MUAC. Health-related quality of life should be taken into consideration in the assessment and treatment of patients with cirrhosis. Older patients, patients with decompensated cirrhosis, and patients with lower MUAC measurements should be given special attention.

## Introduction

Cirrhosis is the end stage of liver disease characterized by irreversible hepatic fibrosis as a result of repeat injury (1). Cirrhosis is an ever-increasing problem. It has become one of the top 10 leading causes of death in the world (2). Cirrhosis contributed to 2.4% of deaths globally in 2017. Sub-Saharan Africa has the highest mortality from cirrhosis (3, 4). Liver cirrhosis is the seventh leading cause of death in Ethiopia, hepatitis B virus, hepatitis C virus, and alcohol consumption are the major causes (5).

Cirrhosis decreases the overall metabolic ability of the liver, leading to different complications (6) including ascites (7), gastrointestinal bleeding (8), encephalopathy (9), loss of energy, and malnutrition (10). These symptoms and metabolic derangements decrease the overall quality of life of patients.

Health-related quality of life (HRQoL) is a concept about an individual’s perception of their physical, social, emotional, and mental well-being. It reflects a patient’s experience with the disease and treatment (11, 12).

The Chronic Liver Disease Questionnaire (CLDQ) is a widely used method for assessing HRQoL in cirrhosis patientsHRQoL in patients with cirrhosis (13). The questionnaire was developed by Younossi and colleagues in 1999. It has been validated and used by researchers worldwide (14).

Measuring HRQoL in patients with cirrhosis provides information about the extent and the effect of the disease on individuals. Additionally, it can be used to improve treatments, health system delivery (15), determining the prognosis, and decision-making (16, 17).

In countries like Ethiopia, there is a high burden of cirrhosis and low treatment capacity, cirrhosis causes socioeconomic, and health burdens among patients, but there is no information relating to HRQoL in patients with cirrhosis. Therefore, this study aimed to assess HRQoL and associated factors among patients with cirrhosis in Ethiopia.

## Methods

### Study design and settings

A cross-sectional study was conducted at Tikur Anbessa Specialized Hospital in Addis Ababa, Ethiopia. The hospital is one of the main tertiary care centers in the country and provides services for referral cases from all over the country. Data were collected from September to December 2021.

### Study population

The study population were all patients with cirrhosis who visited the gastrointestinal unit of Tikur Anbessa Specialized Hospital during the study period. Two hundred twenty-one patients with cirrhosis were included.

### Inclusion and exclusion criteria

All patients (age ≥ 18 years) who were diagnosed with cirrhosis were included. Patients with hepatocellular carcinoma and patients who were unable to communicate were excluded from the study.

### Study variables

The dependent variable was health-related quality of life (HRQoL) assessed with the Chronic liver Disease Questionnaire (CLDQ). The CLDQ has six domains: namely abdominal symptoms (AS), fatigue (FA), systemic symptoms (SS), activity (AA), emotional function (EF), and worry (WO). The independent variables were socio-demographic variables (age, sex, marital status, occupation), and clinical variables (cirrhosis degree, Child-Turcotte-Pugh class, etiology of cirrhosis, MUAC, and serum albumin).

### Data collection procedure and data quality assurance

Data were collected by face-to-face interview by four interns and supervision was done by three residents. The questionnaire was prepared in English and translated to Amharic, then translated back to English to check the consistency. Three-day training was given to the data collectors. Close supervision was conducted by the investigator and supervisors during the data collection and data entry.

Health-related quality of life (HRQoL) was assessed using the CLDQ (chronic liver disease questionnaire), which is disease disease-specific questionnaire developed for use in CLD patients by Younossi and colleagues (13). The questionnaire consists of 29 questions. The response is assessed on a 7-point Likert scale and the responses range from 1 “all of the time” to 7 “none of the time”. A higher response indicates a good level of functioning, while a lower response indicates a low level of functioning. There are six domains in the questionnaire, which include abdominal symptoms, fatigue, systemic symptoms, activity, emotional functioning, and worry.

Ascites grading was done according to the international ascites grading system. Patients with ascites detectable with ultrasound were grouped as grade 1, patients with moderate ascites were grouped as grade 2, and patients with marked abdominal distention were grouped as grade 3 (18).

Scoring for the Child-Turcotte-Pugh classification was done by incorporating values of encephalopathy, ascites, bilirubin, albumin, and international normalized ratio (INR). Patients were grouped into class A, class B, and class C depending on the scores (19).

### Data processing and analysis

Data were coded and entered into Epi Data 4.6.0.2 and analyzed by STATA software version 17m/p. Descriptive statistics with frequency and percentage were used to characterize socio-demographic and clinical variables. Bivariable and multivariable linear regression analyses were conducted, and variables with p-value <0.05 were considered statistically significant. The models showed no influential cases, no multicollinearity, no heteroscedasticity, and residuals were normally distributed showing good fit model.

### Ethics approval and consent to participate

The study was conducted after ethical clearance was obtained from the ethics committee of the Department of Internal Medicine, School of Medicine, Addis Ababa University. The objectives of the study were explained to the participants. Verbal and written consent was obtained from each participant.

## Results

A total of 221 patients with cirrhosis were included in the study. The mean ± SD age was 42.74 ± 12.25. Among the total patients, 52 (23.53%) were female and 169 (76.47) were male. Thirty two (14.48%) patients were single, 180(81.45%) were married while the remaining 9 (4.07%) were divorced or widowed (**Table 1**).

**Table 1:** Summary of socio-demographic characteristics of patients with cirrhosis at Tikur Anbessa Specialized Hospital, Addis Ababa Ethiopia.

Variables	Category	Frequency (%)
Age (years)	18-33	58 (26.24)
	34-49	88 ( 39.82)
	50-65	75 (33.94)
Sex	Female	52 ( 23.53)
	Male	169 ( 76.47)
Marital status	Single	32 (14.48)
	Married	180 (81.45)
	Divorced & widowed	9 (4.07)
Occupation	Farmer	62 (28.05)
	Civil servant	88 (39.82)
	Private worker	46 ( 20.81)
	Other	25 (11.31)

### Clinical characteristics

The mean ± SD of serum albumin was 3.36 ± 0.73. Seventy-one (32.13%) of patients had compensated cirrhosis while the remaining 150 (67.87%) had decompensated cirrhosis. According to Child-Turcotte-Pugh classification 95 (42.99%) of patients were class A, 102 (46.15%) were class B, and 24(10.86%) were class C. Regarding ascites 110 (49.77%) patients had grade 1 ascites, 87 (39.37%) had grade 2 ascites, and the remaining 24 (10.86%) had grade 3 ascites (**Table 2**).

**Table 2:** Clinical characteristics of cirrhosis patients at Tikur Anbessa Specialized Hospital Addis Ababa Ethiopia.

Variable	Category	Frequency (percentage)
Degree of cirrhosis	Compensated	71 (32.13)
	Decompensated	150 ( 67.87)
CTP classification	Class A	95 (42.99)
	Class B	102 (46.15)
	Class C	24 (10.86)
Ascites	Grade 1	110 ( 49.77)
	Grade 2	87 (39.37)
	Grade 3	24 (10.86)
Serum albumin	Mean ± SD	3.36±0.73
MUAC (cm)	Mean ± SD	24.10± 2.32
BMI( kg/m^2^)	Mean ± SD	20.22±2.58

BMI, Body mass index; CTP, Child-Turcotte-Pugh classification; MUAC, mid-upper arm circumference.

### Quality of life using the chronic liver disease questionnaire

Health Related Quality of life (HRQoL) was assessed using the Chronic Liver Disease Questionnaire (CLDQ) which has 29 items with six domains including abdominal symptoms, fatigue, systemic symptoms, activity, emotional functioning, and worry. The response was scored 0-7. A higher score indicates a higher level of functioning and good quality of life while a lower score indicates lower quality of life.

Among the domains lower mean value was observed in the worry domain Mean ± SD (3.60± 1.21) while a higher mean score was observed in the systemic symptoms domain Mean ± SD (5.63± 1.13) (**Table 3**).

**Table 3:** Mean score of CLDQ score and domains of patients with cirrhosis at Tikur Anbessa Specialized Hospital.

CLDQ domains	Mean ± SD	Minimum	Maximum
Mean CLDQ score (global rating )	4.55 **±** 1.25	2.1	7
Abdominal symptoms	3.97 ± 1.74	1	7
Fatigue	4.30 ± 1.69	1.4	7
Systemic symptoms	5.63± 1.13	1.8	7
Activity	4.67± 1.93	1	7
Emotional function	4.80± 1.23	2.1	7
Worry	3.60± 1.21	1.6	7

CLDQ, Chronic liver Disease Questionnaire.

### Health-related quality of life among patients with compensated and decompensated cirrhosis

Independent samples t-test was used to compare differences in mean HRQoL score, and mean scores in HRQoL domains including; abdominal symptoms, fatigue, systemic symptoms, activity, emotional functioning, and worry between patients with decompensated cirrhosis and compensated cirrhosis. Patients with decompensated cirrhosis scored significantly lower in the mean CLDQ score and all the domains (**Table 4**).

**Table 4:** Independent samples t-test of mean CLDQ and domains among patients with compensated and decompensated cirrhosis.

Variables	Compensated cirrhosis (mean±SD)	Decompensated cirrhosis (mean±SD)	P=value
Mean CLDQ score	5.44±0.98	4.12±1.14	P<0.001
Abdominal symptoms	4.99±1.51	3.49±1.64	P<0.001
Fatigue	5.56±1.21	3.70±1.57	P<0.001
Systemic symptoms	6.10±0.99	5.37±1.13	P<0.001
Activity	5.87±1.36	4.05±1.88	P<0.001
Emotional function	5.55±1.05	4.43±1.16	P<0.001
Worry	4.52±1.14	3.20±1.06	P<0.001

### Factors associated with health-related quality of life scores

A bivariable linear regression analysis was performed to obtain candidate variables for multivariable linear regression analysis. Variables with a p-value of less than 0.25 were included in the multivariable linear regression analysis. These variables included: Age, Sex, Serum albumin, MUAC, CTP score, degree of cirrhosis, and etiology of cirrhosis.

In multivariable linear regression analysis MUAC (Std.β.coff = 0.12, 95% CI (0.06, 0.18), age ≥ 50 (Std.β.coff = -0.38, 95% CI (-0.68,-0.09), Child- Turcotte Pugh (CTP) class B (Std.β.coff = -0.92, 95% CI (-1.28, -0.55), CTP class C (Std.β.coff = -1.69, 95% CI (-2.30,-1.07) were significantly associated with quality of life score (**Table 5**).

**Table 5:** Multivariable linear regression analysis on factors associated with quality of life score.

Variables	Category	Total number/ Mean ± SD	Unstandardized β (95% CI)	Standardized β (95% CI)	P-value
Age years	20-34	60	1	1	
	35-49	86	-0.08 (-0.49,0.32)	0.19 (-0.07,0.46)	0.14
	≥50	75	-0.60 (-1.02,-0.18)	**-0.38 (-0.68,-0.09)**	**0.01**
Sex	Female	52	1	1	
	Male	169	-0.04 (-0.44,0.35)	-0.10 (-0.37, 0.17)	0.46
MUAC (cm)	mean±SD	24.1±2.32	0.31 (0.25, 0.37)	**0.12 (0.06, 0.18)**	**<0.001**
Serum Albumin (gm/dl)	mean±SD	3.36±0.73	1.10 (0.92, 1.28)	0.13 (-0.10, 0.38)	0.26
Degree of cirrhosis	Compensated	71	1	1	
	Decompensated	150	-1.30 (-1.62,-0.98)	-**0.40 (-0.67,** -**0.13)**	**<0.01**
CTP class	Class A	95	1	1	
	Class B	102	-1.86 (-1.79,-1.33)	**-0.92 (-1.28,** -**0.55)**	**<0.001**
	Class C	24	-2.78 (-3.15,-2.40)	**-1.69 (-2.30, -1.07)**	**<0.001**
Etiology of cirrhosis	Hepatitis B	86	1		
	Hepatitis C	33	-0.62 (-1.13, -0.12)	-0.08 (-0.29, 0.45)	0.67
	Alcohol related	66	-0.24 (-0.64, 0.15)	-0.03 (-0.31, 0.23)	0.77
	Other	36	0.53 (0.03, 1.03)	-0.01 (-0.35, 0.32)	0.94

CTP class, Child-Turcotte-Pugh classification; MUAC, Mid Upper Arm Circumference.

Bold values indicate significantly associated values.

## Discussion

Patients with liver cirrhosis face different complications that reduce their quality of life. HRQoL is important to reflect patients perception on disease progression and treatment. The CLDQ is a disease-specific questionnaire to assess HRQoL in patients with cirrhosis (13).

In this study age is one of the significant predictors of quality of life. Patients older than 50 had worse quality of life. In line with the current study, studies conducted among community-dwelling adults in Spain and China, as well as a study among heart failure patients in Ethiopia, have demonstrated that older adults have lower HRQoL compared to younger adults (20–22). However, there are conflicting findings regarding the impact of age on HRQoL in patients with CLD. A study conducted among CLD patients in Thailand found that older age negatively affects HRQoL (23), while a study conducted in Canada showed younger age negatively affects HRQoL (24). Meanwhile, studies conducted in Nepal (25) and Germany (26) showed no association between age and HRQoL. Methodological differences and use of different tools may be the reason for the discrepancy of results among the studies. According to studies, showing negative impact of aging on HRQoL, aging by itself lowers physical and mental function which decreases the ability to perform daily activities thereby lowering the overall quality of life (27).

In this study patients with Child-Turcotte-Pugh (CTP) score B and C have lower quality of life sores. This is in line with other articles conducted in India (28) Spain (29) and Pakistan (30). Different reports have shown that the severity of disease influences HRQOL including heart failure (21), Lupus (31), and Cancer (32). As the severity of the disease increases, symptoms and complications from the disease cause, limitations in physical, mental, and emotional function which lowers the overall quality of life (33).

Findings from our study indicate that as MUAC increases mean quality of life score also increases. MUAC is a simple non-invasive anthropometric indicator widely used to assess malnutrition among children and adults globally. Studies have shown that MUAC measurement can detect malnutrition with good accuracy similar to Triceps skin fold thickness and Middle arm Muscle circumference (34). MUAC has been used to measure appendicular muscle mass, sarcopenia, and malnutrition in patients with cirrhosis (10, 35). Reports have shown that malnutrition lowers HRQoL in patients with cirrhosis (36).

## Limitations

One limitation of this study is the lack of control groups, the researchers couldn’t incorporate control groups due to budget constraints. Additionally, since the data was collected at a tertiary hospital, the results may not be generalizable to all patients with cirrhosis.

## Conclusion

In this study, mean HRQoL score was significantly associated with age, CTP classification, and MUAC. Routine assessment HRQoL should be considered in patients with cirrhosis. Older patients, patients with advanced cirrhosis, and patients with lower MUAC scores should be given special attention.

## Data Availability

The original contributions presented in the study are included in the article/supplementary material. Further inquiries can be directed to the corresponding author.
